# Off-line measuring sampling data identification parameters for digital twins mirroring load modelling and stability analysis

**DOI:** 10.1038/s41598-023-31451-9

**Published:** 2023-03-21

**Authors:** Javier Urquizo, Nathalie Ramirez, Dietmar Sanchez, Juan Plazarte

**Affiliations:** 1 Escuela Superior Politécnica del Litoral, FIEC, Campus Gustavo Galindo Km. 30.5 Vía Perimetral, PO Box 5863, Guayaquil, Ecuador; 2 Departamento de Expansión de la Transmisión, CELEC/Transelectric, Avenida 6 de Diciembre y Orellana, Quito, Ecuador

**Keywords:** Energy grids and networks, Power distribution, Power stations

## Abstract

Currently, the methods used to represent loads do not differ between the characteristics that compose them or the nature of these. Therefore, the purpose of this research is to develop digital twins mirroring load models that can be used for more precise studies on power-flows and stability within the National Transmission Grid (NTG). Off-line sampling data of different electric measurements have been used in six substations of the Guayaquil (Ecuador) area. These values were organized by statistical methods and by time periods, to determine the parameters that make up the static load model. Dynamic models are also constructed for the same six substations using the analysis of current and voltage signals obtained from the substations. All data is organized to show a digital twin mirroring visual representation of the disturbances that may occur in the substation buses. A more accurate description of the static and dynamic responses can be obtained by replacing the general model that is currently used by engineers and planners with off-line sampling data. Digital twins help the electric utility businesses gather, visualise, and contextualise data from different sources, and enable to act on data, and to understand what-if modelling stability scenarios.

## Introduction

Load modelling is important for energy system analysis, planning, and control. This study demonstrates the importance of accurate load representations in assessing voltage stability^1,2^. Load modelling consists of two major steps. First select the load model structure and then use a component or measurement-based approach to identify the load model parameters. Component-based is based on an understanding of the physical behaviour of the load and the mathematical relationships that describe how the load device works^3^. However, such information is not always available, leaving practical digital twin mirroring methods for measurement-based modelling approaches^4^ of physical energy systems. A physical energy system refers to the ‘real world’ environmental space in which physical entities exist. Aspects of these entities are measured and fed into a digital twin mirroring environment to ensure an accurate virtual environment for simulation, fine-tuning, and/or making decisions^5^. A simulation recreates what happens to a power system, while a digital twin recreates and introduces a concrete realization for mirroring what happens to a specific power system in the real-time world. Load modelling consists of two main steps: first selecting a load model structure, and second identifying the load model parameters using component or measurement-based approaches. Component-based is based on the understanding of physical behaviours of loads and mathematical relations that describe the functioning of load devices^3^. However, obtaining such information is not always possible, which leaves a practical digital twin mirroring method of measurement-based modelling approach^4^ of the physical power system. This paper uses a measurement-based modelling approach. We argue that engineering and operations require measurement-based methods, rather than expert centred tools, to support systems that continuously interact with physical and digital environments through simulation^1,2,6^.

The Ecuadorian Government, through the Electricity Corporation of Ecuador (CELEC EP) implemented the Transmission Operation Centre (TOC) for substations and transmission lines operated by the Transelectric Business Unit. TOC allows real time measures of the National Transmission Grid (NTG) operation in a safe and reliable way. TOC collects measurements from data acquisition equipment that would allow this research to derive load characteristics. However, a developed model at one network location may not be applicable to other locations.

### Static load model

Load modelling is a mathematical representation of the relationship between power and voltage on the load bus^7^. Load models can be divided into two main categories: static models and dynamic models^8^. A static load model represents the load as a time-invariant function of voltage and frequency. The load behaviour at any point in time is given as a function of voltage and frequency. However, excessive frequency deviation in the system is unacceptable, so automatic frequency control is used to maintain acceptable frequency during faults. In addition, spontaneous load changes in power systems introduce minute-to-minute fluctuations. This requires some form of frequency control that most systems use. In this study, frequency dependent components of the load model are ignored.

Static load models are exponential load models and polynomial load models. The ZIP model is a combination of constant impedance (Z), constant current (I), and constant power (P) models. The polynomial models are a general form that encompasses the ZIP and exponential models. The ZIP model is a special case of this model, obtained for exponents of 2, 1, and 0 respectively, so the first summand corresponds to the constant impedance model, the second to the constant current model, and the third corresponds to constant power. The model used by PF corresponds to a polynomial model. In this study, we followed the CELEC criteria for ZIP model selection. The characteristic equations for the static ZIP model are shown in Eqs. (1) and (2):

1 $$  P = P_{0} \left[ {a_{1} \left( {\frac{V}{{V_{0} }}} \right)^{2}  + a_{2} \left( {\frac{V}{{V_{0} }}} \right) + a_{3} } \right] $$

2 $$Q={Q}_{0}\left[{a}_{4}{\left(\frac{V}{{V}_{0}}\right)}^{2}+{a}_{5}\left(\frac{V}{{V}_{0}}\right)+{a}_{6}\right]$$

where *V*_*0*_ is the voltage, *P*_*0*_ is the active power and *Q*_*0*_ is the reactive power. A static model is time-independent and represents the relationship between power (active or reactive) at a particular point in time and voltage and frequency at the same point in time. These are the values at the initial conditions of the system for the study, and the coefficients *a*_*1*_ through *a*_*6*_ are the model parameters.

### Dynamic load model

Dynamic load models represent loads using current and previous states of voltage and frequency^9^. In general, according to the literature, the differences in results between dynamic load models depend on the results of various field measurements, and their intended use appears to be small. For example, Lin et al.^10^ use a technique based on the concept of confidence intervals to validate the developed high order loading model structure. Ju et al.^11^ present both dynamic and static load characteristics and provide an approach to the parameters using genetic algorithms. Liang et al.^12^ have developed a measurement-based model to consider the behaviour of voltage regulation devices, particularly switched distribution capacitor banks and substation on-load tap-changer transformers, and Karlsson and Hill^13^ describe an exponential dynamic loading model. In this study, we followed the CELEC criteria for choosing exponential dynamic loading models described in Eqs. (3) and (4):

3 $${T}_{p}\frac{d{P}_{r}}{dt} + {P}_{r}={{P}_{0}\left(\frac{U}{{U}_{0}}\right)}^{{\alpha }_{s}}+{{P}_{0}\left(\frac{U}{{U}_{0}}\right)}^{{\alpha }_{t}}$$

4 $$Pl=Pr+Po{\left(\frac{U}{Uo}\right)}^{{\alpha }_{t}}$$

where *U*_*o*_ and *P*_*o*_ are the voltage and power consumption before the voltage change. *P*_*r*_ is the active power recovery, *P*_*l*_ is the total active power response, *T*_*p*_ is the active load recovery time constant, α_t_ is the transient active load voltage dependence, and α_s_ is the steady-state active load voltage dependence^14^. A similar formula applies to reactive power. The load is described by a time constant, and transient and steady-state load voltage dependent parameters. *T*_*p*_ represents the time it takes for the power recovery to reach 63% of its final value, and the steady-state load voltage dependence represents how much load has recovered after recovery i.e., zero means a fully restored load, another value represents a partially restored load. It can also take negative values if there is an overshoot in the load response. Transient load voltage dependence describes the load response to disturbance i.e., zero means constant power load response, value one means constant current load response, value two means constant impedance load response^15^. Considering that the dynamic behaviour of the load follows a first-order differential equation, the power versus voltage PV follows an exponential model.

### Stability analysis

The power grid is not a static system as it is subject to various disturbances on the load and generator side of the grid. These dynamic unbalances the system. After a disturbance, the network can rebalance and continue operation only if the operating point exceeds the maximum load point. Otherwise, the voltage stability will be lost. Therefore, the power-voltage curve (PV) provides important information about the stable operating point of the system. In addition, system operators know how much additional system load they can handle without losing stability. Voltage stability is further classified as small disturbance and large disturbance voltage stability^16^. In small disturbance voltage stable system, voltage restores near to the pre-disturbance stable state after the system is subjected to small disturbances. In the large disturbance voltage stable system, the system voltage reaches a post-disturbance equilibrium state, after large disturbances caused due to various types of faults in the system and contingencies.

Measurement-based techniques have the obvious advantage of obtaining data directly from the actual system. However, application of data gathered at one substation to load models for other substations may only be possible if the loads are very similar. One way of overcoming this disadvantage is to use measurements made on feeders supplying specific classes of loads, that is, residential, commercial, industrial, etc., therefore, to obtain typical load class characteristics.

Once the proposed load model is defined in the selected region, the system characteristics, namely the power flow results before and after the load variation, are compared at different points in the developed model Guayaquil area. Its consumption is determined from actual measurements at substations^17^. The load model described in this document replaces all loads in the system. Simulations are done in the DigSILENT PowerFactory Simulator (PF). Static and dynamic models of each substation are calculated and used as inputs for the PF tool’s power flow analysis simulations so that conclusions about system stability can be drawn^18,19^.

## Load model methodology

**Figure 1:**
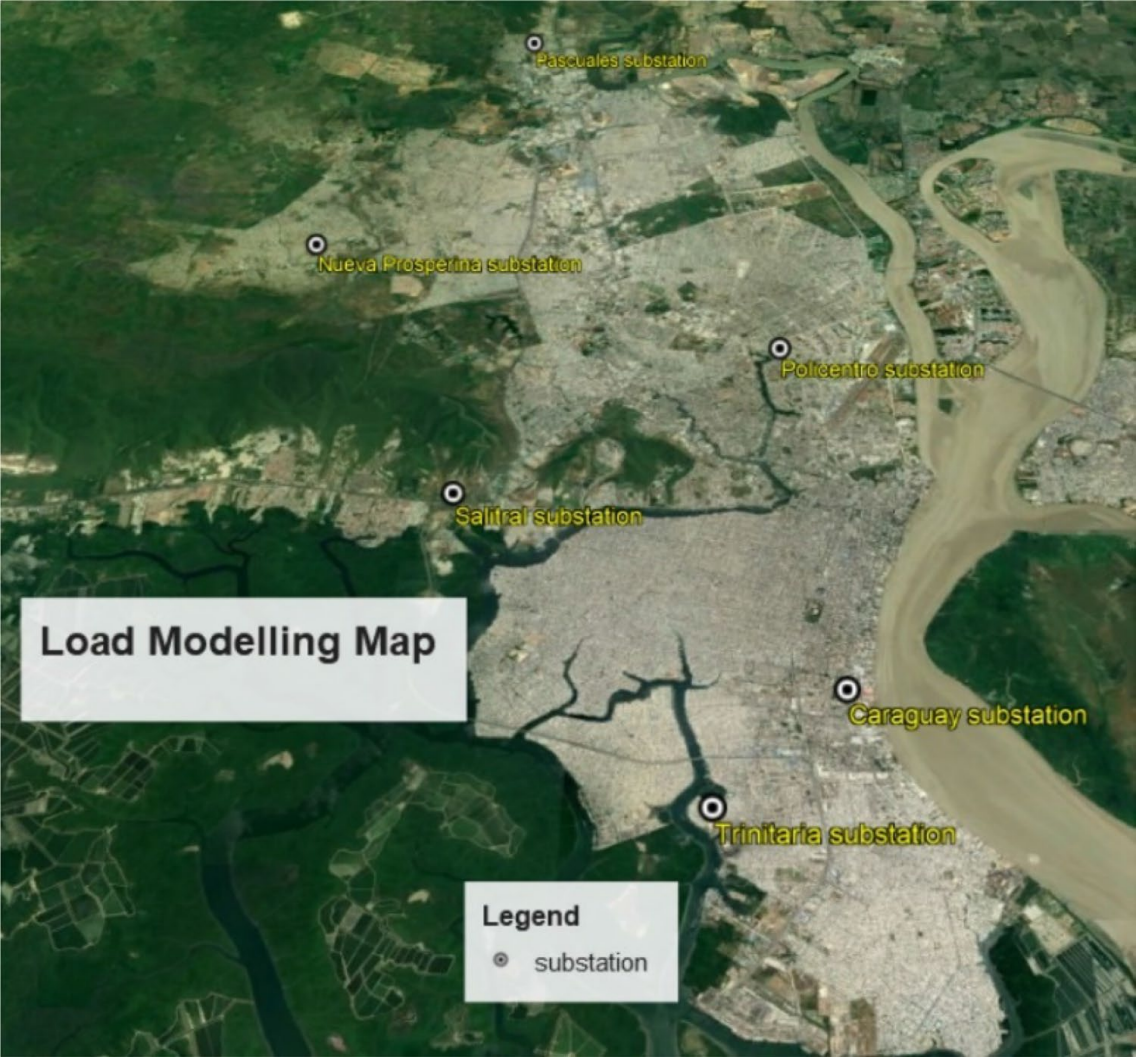
Google Earth oblique image of Translectric substations entry points to Guayaquil^20^.

Off-line measurements are from six Transelectric substations (Policentro, Pascuales, Caraguay, Nueva Prosperina, Salitral and Trinitaria) (see Fig. 1). These substations provide an entrance for Guayaquil’s energy needs and an exit from the national grid.

### Step-by-step static load procedure

The load class data for Guayaquil is grouped into industrial, residential, and commercial load data. Industrial loads correspond to industrial land along the northwest side of the city. Heavy industry includes cement factories and breweries. Thermal energy demand in cement plants can account for about 20–25% of the total cost of cement production^21^. Brewing is a heat and electricity intensive process^22^. Most of the load on homes is home appliances related to daily life but cooling in winter also accounts for a large proportion. Commercial loads make up the majority of air conditioning and discharge lamps.

Different load components generally form different load classes. A load with ‘fast dynamic’ electrical and mechanical properties that does not exhibit significant discontinuities or delays in response to voltage disturbances such as incandescent lamps and loads with ‘slow’ characteristics such as electric heating.

Mercury vapour, sodium vapour, and fluorescent loads form the main lighting in industrial and street lighting and make up the bulk of the load composition in commercial areas. They are very sensitive to voltage fluctuations. A common application for residential and commercial engines is air conditioning and refrigeration compressor loads. Pumps, fans, and compressors make up an important part of industrial engines.

For the static load model of ZIP, we need to obtain the parameters observed in the Eqs. (1) and (2). Load model parameters are estimated by fitting all substation data to a ZIP model. Determining static load model parameters from sudden step changes is relatively straightforward. Since the ZIP model is a linear combination of impedance, power, and constant current, the coefficients *a*_*1*_, *a*_*2*_, *a*_*3*_, *a*_*4*_, *a*_*5*_ and *a*_*6*_ can be obtained as:

1. Off-line CELEC measurements show the energy consumption of six substations. The energy to power conversion is in the Eq. (5). This formula takes into account the consumption data migration period of 1 month.
  5 $$MontlyPower \,\left[{\mathrm{MW}}\right]=Energy \,\left[{{\mathrm{KWh}}}\right]\times \frac{1\, {\text{day}} }{\mathrm{a}}{24\,{\mathrm {h}}}\times \frac{1\,{\mathrm{month}}}{30\,{\mathrm{days}}}$$
2. Using the power factor data for each transformer, the reactive power can be determined using the known power triangle. The reactive power value *Q* is equal to *P × *tan*Φ* supplied by each distribution transformer in the selected substation.
3. In the ZIP model, the constant power components represent the industrial loads in the system, so the values *a*_*3*_ and *a*_*6*_, the constant current represents the commercial loads in the system, so the values of *a*_*2*_ and *a*_*5*_, and finally the constant impedance represents the residential load in the system, so, the values of *a*_*1*_ and *a*_*4*_^23–25^.
4. Various algorithms can be used to obtain the ZIP coefficients based on the measured voltage–power values. In this research, the least squares method (LSM) is used due to its simplicity. For the values of *V*, we use least squares method on the historical data for the selected time period to reduce the data and obtain the values used to determine the model. For the value of *V*_*0*_, this research uses the nominal voltage of the substation. The objective function is denoted by Eq. (6):
  6 $$\uplambda =\sum_{i=1}^{n}[Zp (Vi/V0 )^2+Ip (Vi/V0 )+Pp - Pi/P0 ]$$

where *V*_*i*_ and *P*_*i*_ are the voltage and active power measurements, respectively. To solve a least squares problem, the partial derivative of the objective function for each variable must be zero. Therefore, we have three equations for the three ZIP coefficients. The solution is achieved by solving these three equations. The solution matrix is given by Sadegni and Abdollahi^26^. The values of *P*_*0*_ and *Q*_*0*_ correspond to those by the substation under normal operating conditions.

### Step-by-step dynamic load procedure

In developing of the dynamic load model, historical Off-data voltages, and currents were used at each of the six substations. To obtain a dynamic model, the perturbations occurring in the system are analysed from a selection of observed curve sections^27^.

Once a section is selected for analysis, the three points at which voltage disturbances are observed are taken from the diagram. The first analysis point is obtained with stable operation i.e., just moments before the occurrence of the disturbance. The second point belongs to the section of the diagram where disturbances or voltage drops are shown i.e., just at the moment where the voltage in the bus begins to decay, that is, in the range where voltage drop occurs. Finally, the third point is obtained when the system starts to stabilize, and the voltage values are within the normal operating range^19^. The procedure is described with the following steps.

**Figure 2:**
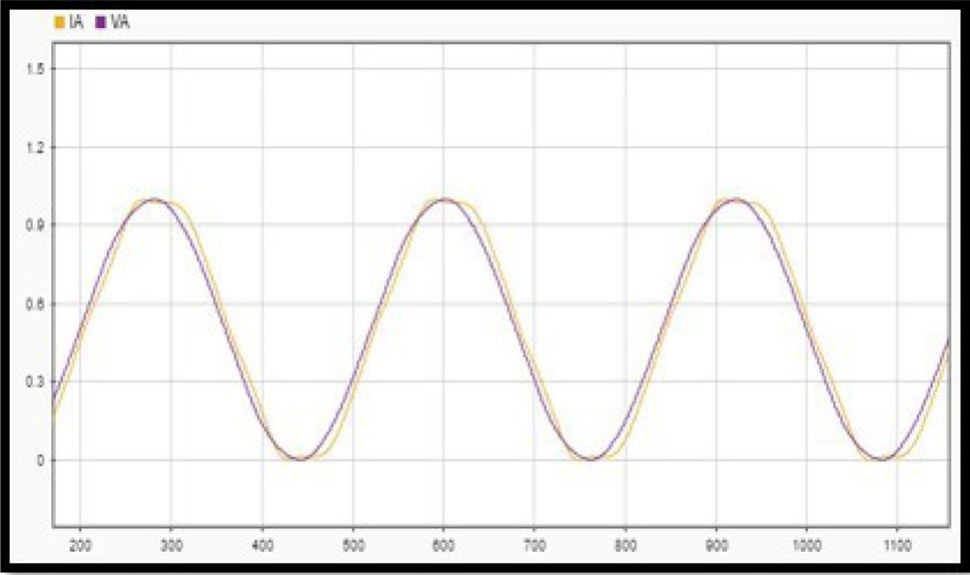
Superposition of voltage and current signals at the Policentro substation.

1. Data must be organized for proper analysis. A high sampling frequency divides millions of data into intervals.
2. After the data is converted into the appropriate format, it is displayed in graphs and analysed for errors. This is done using the MATLAB Signal Analyser Tool (SAnT). The tool records the 192,000 points for each file and undergoes a visual inspection to detect erroneous data. This can be seen in Fig. 2 using Policentro bus as an example.

Figure 2 shows the superposition of the voltage and current signals, the data of which was acquired at a high sampling frequency. Signals to outputs of all six substations under investigation follow the same procedure.

3. Find the phase difference between the voltage sine wave and the current sine wave for each phase. This is done using SAnT, as shown in Fig. 3, taking into account the temporary space that exists between each data acquisition.

**Figure 3:**
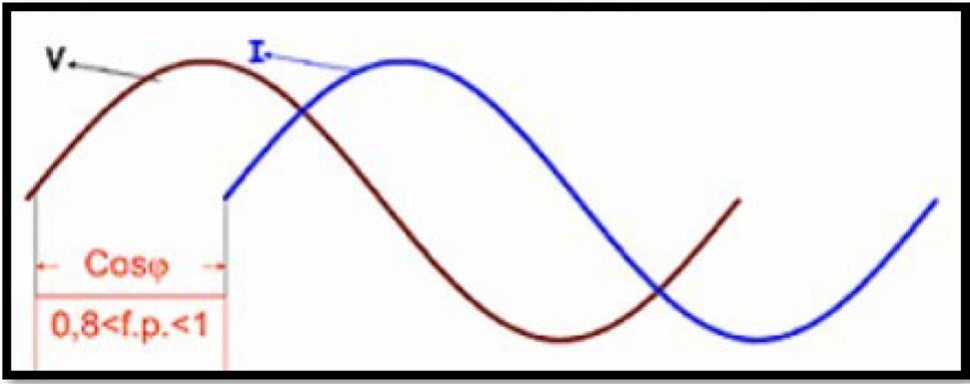
Phase difference between the voltage and current waves.
  The power factor can be determined from the phase shift shown in Fig. 3 via the angular difference between the two signals. For Policentro substation 192,000 values are obtained from a time range of 10 s. The time interval between one data and the next is given by Eq. (7):
  7 $$\mathrm{Data\, intervals }= (\mathrm{Sampling \,time})/ (\mathrm{Number \,of \,data})$$
  This number corresponds to a value representing each subdivision of the abscissa (x-axis) of the plane in which the voltage and current waves are plotted. Therefore, to obtain the phase shift, start by placing one of the cursors at the intersection through the origin of the current wave ordinate (Y-axis) and the other at the origin of the voltage wave ordinate. Multiply the resulting distance value by the value obtained for the data acquisition interval. This way we get the offset as a unit of time (s).
  Knowing that Ecuador has a frequency value of 60 Hertz (60 cycles per second), the period of the wave is obtained using the Eq. (8). This period constitutes a 360° path in the sine wave. Compute X from Eq. (8), where N is given in seconds:
  8 $$\mathrm{T}=1/\mathrm{f};\mathrm{ X}=(\mathrm{N}\times 360)/ (0.0167)$$
4. Once the phase shift is obtained, proceed to the root mean square (RMS) values of the voltage, current, and power waveforms. Since the sinusoid could not show the glitches that the system could represent, we need the RMS value graph wave.

## Static polynomial load model results

The polynomial (ZIP) load model represents the load with a mix of the three types of loads. This model is expressed as shown by Eqs. (1) and (2). Here *a*_*1*_ = *Z*_*p*_, *a*_*2*_ = *I*_*p*_, *a*_*3*_ = *P*_*p*_, *a*_*4*_ = *Z*_*q*_, *a*_*5*_ = *I*_*q*_ and *a*_*6*_ = *P*_*q*_ are the model parameters representing the percentages of constant impedance, constant current, and constant power load. Additionally, terms that reflect the frequency dependency in the model are ignored.

The historical data available from the six substations for load modelling are: (1) total energy supplied by the substation, (2) total energy supplied to residential customers, (3) total energy supplied to industrial customers, (4) total energy supplied to commercial customers, (5) total energy loss, (6) total energy supplied to other customers, (7) distribution transformers connected to the substation power factor, (8) energy supplied to each consumer type for each distribution transformer connected to the substation, (9) historical voltage data.

Following the step-by-step procedure described in “Load model methodology”, “Policentro substation static load model” through “Trinitatia substation static load model” shows tables for the static load coefficients for the Active Power, Reactive power, maximum, minimum, and average voltage of the buses, and the Active power and Reactive power expressions.

### Policentro substation static load model

The static load model coefficients for the Policentro substation ZIP are given in the left of Table 1. The 69 kV voltage bus is shown in Table 1 on the right. Equations (9) and (10) are the ZIP static load model for Policentro substation:

**Table 1:** Policentro ZIP static load model coefficients and voltage bus values.

Model	APC	RPC	Model	APC
Constant impedance	0.64507532	0.58311912	Maximum Voltage	1.02396314
Constant current	0.3485863	0.41064004	Minimum Voltage	0.98920352
Constant power	0.00633839	0.00624085	Average Voltage	1.00596076

9 $$P={P}_{0}\left[0.64507532{\left(\frac{V}{{V}_{0}}\right)}^{2}+0.3485863\left(\frac{V}{{V}_{0}}\right)+0.00633839\right]$$

10 $$Q={Q}_{0}\left[0.58311912{\left(\frac{V}{{V}_{0}}\right)}^{2}+0.41064004\left(\frac{V}{{V}_{0}}\right)+0.0062408\right]$$

**Figure 4:**
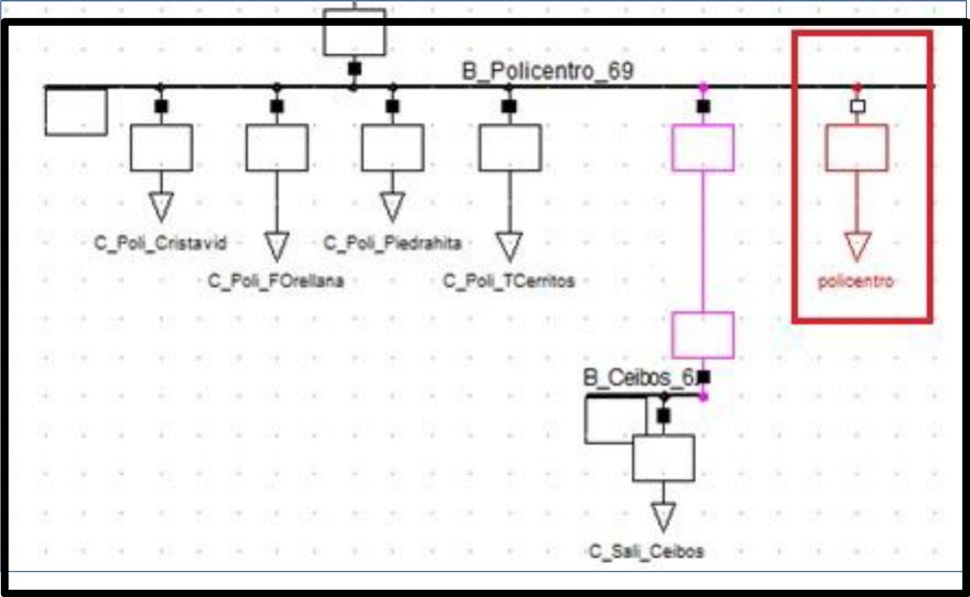
Policentro static (generic) load model representation.

Figure 4 shows the PF Policentro substation bus with newly entered ‘general type’ load parameters. This corresponds to the static load model (see red box). For power flows, the frequency is constant and equal to the nominal value, so only the dependence of power demand on voltage needs to be considered. This is due to the approach to the power flow problem as the reference generator (connected to the balance node) is calculated such that the total system power balance is always met.

The bus voltage magnitudes and angles after modelling the actual voltage values in [pu] of the substation are given in Table 2. Left without load model representation, right with load model representation.

**Table 2:** Changes in magnitude and angle before (left) and after (right) the Policentro static load model.

	Voltage kV	Angle		Voltage kV	Angle
Policentro 69	69.1	− 25.7	Policentro 69	69.3	19.3
Policentro 138	138.6	− 22.1	Policentro 138	143.6	21.4
Pascuales 138	140	− 21.1	Pascuales 138	144.5	22
Pascuales 230	229.6	− 17.5	Pascuales 230	235.8	23.3

### Pascuales substation static load model

The coefficients of the static load model for the Pascuales substation are given in the Table 3 on the left. The 69 kV voltage bus is shown in Table 3 on the right. Equations (11) and (12) are the ZIP static load models for the Pascuales substation:

**Table 3:** Pascuales ZIP static load model coefficients and Voltage bus values.

Model	Load	P		Q	
		Power	Coeff	Power	Coeff
Constant impedance	Resident, other and losses	49.2205	0.60725	9.1082	0.6016
Constant current	Commerce	11.9929	0.14796	2.2345	0.1476
Constant power	Industrial	19.8409	0.24478	3.7953	0.2507

11 $$P={P}_{0}\left[0.607253{\left(\frac{V}{{V}_{0}}\right)}^{2}+0.1479610\left(\frac{V}{{V}_{0}}\right)+0.24478\right]$$

12 $$Q={Q}_{0}\left[0.601675{\left(\frac{V}{{V}_{0}}\right)}^{2}+0.147607\left(\frac{V}{{V}_{0}}\right)+0.250716\right]$$

**Figure 5:**
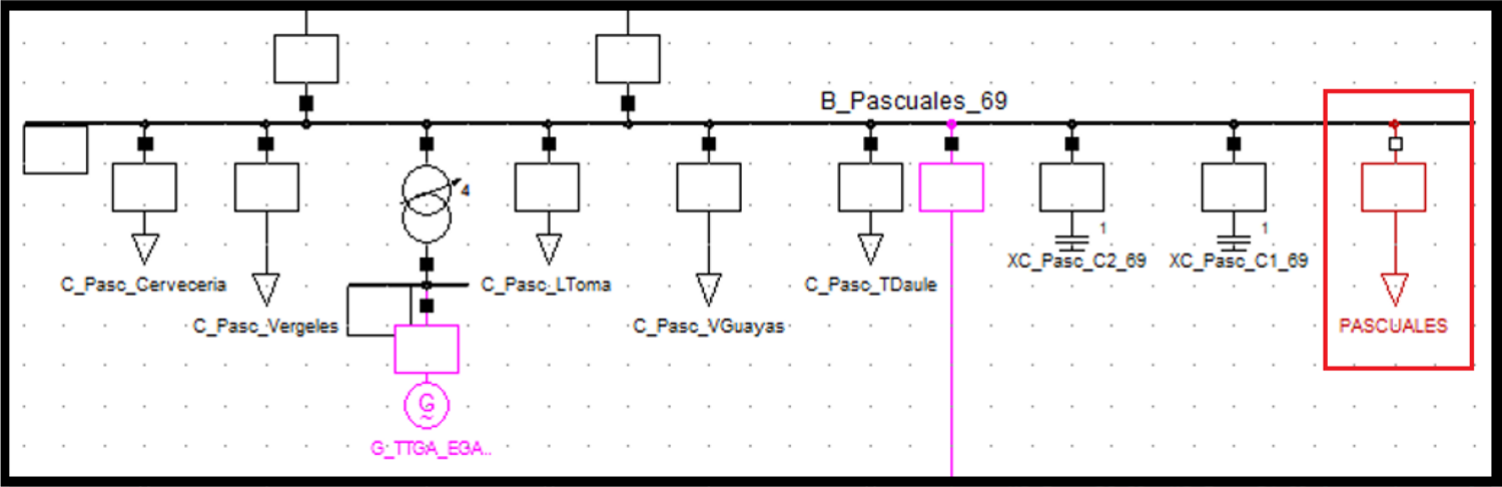
Pascuales static (general) load model representation.

Figure 5 shows the PF Pascuales substation bus with new ‘general type’ load parameters entered according to the static load model (see red box).

The magnitude and angle of the voltage on the bus after entering the model with the real voltage value in [pu] of the substation are given in Table 4. Left without load model representation, right with load model representation.

**Table 4:** Magnitude and angle variation before (left) and after (right) entering Pascuales static load model.

	Voltage kV	Angle		Voltage kV	Angle
Pascuales 69	69.1	− 21.8	Policentro 69	69.2	19.3
Pascuales 138	140	− 19.9	Pascuales 138	144.5	22
Pascuales 230	229.6	− 17.5	Pascuales 230	235.8	23.3

### Caraguay substation static load model

The static load model coefficients for the Caraguay ZIP substation are given in the left Table 5. The 69 kV voltage bus is shown in Table 5 on the right. Equations (13) and (14) are the ZIP static load models for Caraguay substation:

**Table 5:** Caraguay ZIP static load model coefficients and voltage bus values.

Model	APC	RPC	Model	APC
Constant Impedance	0.6572602	0.64173702	Maximum Voltage	1.0317837
Constant Current	0.3078377	0.31563123	Minimum Voltage	0.98229019
Constant Power	0.03490197	0.0426317	Average Voltage	1.00726942

13 $$P={P}_{0}\left[0.657260{\left(\frac{V}{{V}_{0}}\right)}^{2}+0..307837\left(\frac{V}{{V}_{0}}\right)+0.03490\right]$$

14 $$Q={Q}_{0}\left[0.641737{\left(\frac{V}{{V}_{0}}\right)}^{2}+0.315631\left(\frac{V}{{V}_{0}}\right)+0.042631\right]$$

**Figure 6:**
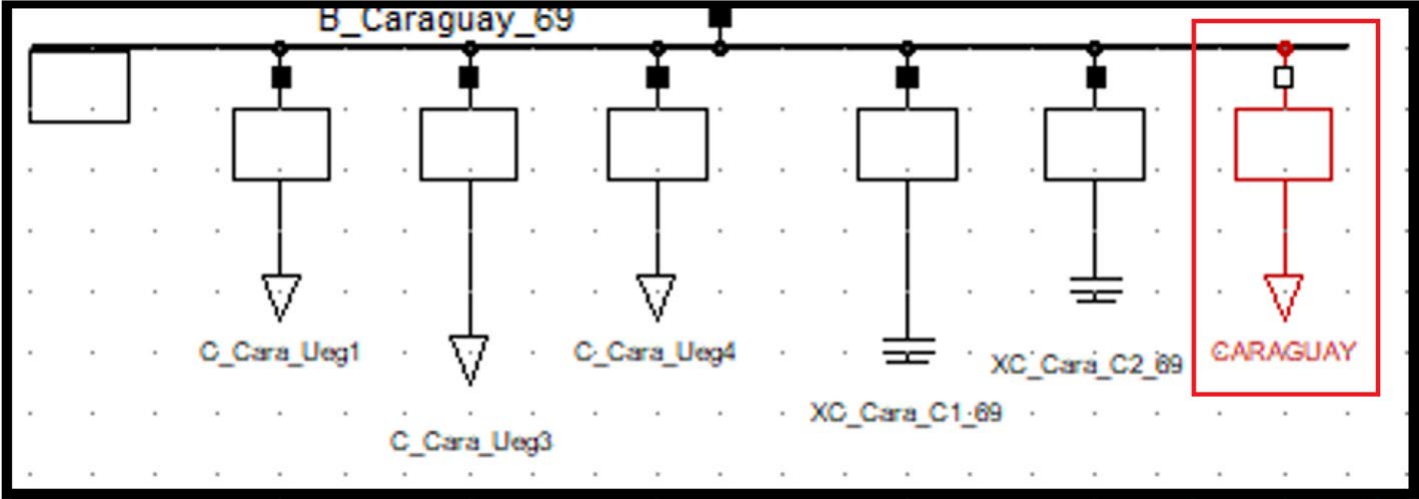
Caraguay static (general) load model representation.

Figure 6 shows the PF Caraguay substation bus with the new ‘general type’ load parameters entered. This corresponds to the static load model (see red box).

The magnitude and angle of voltage in the buses, after entering the model with the real voltage value in [pu] in the substation are shown in Table 6. On the left without the load model representation and on the right with load model representation.

**Table 6:** Magnitude and angle variation before (left) and after (right) entering Caraguay static load model.

	Voltage kV	Angle		Voltage kV	Angle
Caraguay 69	68.9	− 27.8	Caraguay 69	69.2	2
Caraguay 138	139.3	− 24.6	Caraguay 138	146.3	3.4
Escusas 138	139.9	− 23.9	Esclusas 138	146.3	3.4
Esclusas 230	228.7	− 19.4	Esclusas 230	234.2	5.3

### Nueva Prosperina substation static load model

The static load model coefficients for the Nueva Prosperina ZIP substation are shown in Table 7 on the left. The 69 kV voltage bus is shown in Table 7 on the right. Equations (15) and (16) are the ZIP static load models for the Nueva Prosperina substation.

**Table 7:** Nueva Prosperina ZIP static load model coefficients and Voltage bus values.

Model	APC	RPC	Model	APC
Constant Impedance	0.89632974	0.908389005	Maximum Voltage	1.03174537
Constant Current	0.081869057	0.075131368	Minimum Voltage	0.97995426
Constant Power	0.021801203	0.016479628	Average Voltage	1.00733375

15 $$P={P}_{0}\left[0.896329{\left(\frac{V}{{V}_{0}}\right)}^{2}+0..081869\left(\frac{V}{{V}_{0}}\right)+0.021801\right]$$

16 $$Q={Q}_{0}\left[0.908389{\left(\frac{V}{{V}_{0}}\right)}^{2}+0.0751313\left(\frac{V}{{V}_{0}}\right)+0.016479\right]$$

**Figure 7:**
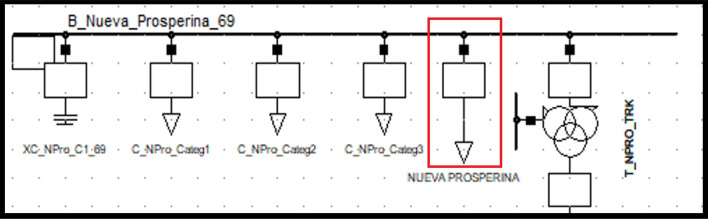
Nueva Prosperina static (general) load model representation.

Figure 7 shows the PF Nueva Prosperina substation bus populated with the new ‘general type’ load parameters corresponding to the static load model (see red box).

The magnitude and angle of the bus voltage after entering the model at the actual value [pu] of the substation voltage are given in Table 8. Left without the load model representation, right with load model representation.

**Table 8:** Magnitude and angle variation before (left) and after (right) entering Nueva Prosperina static load model.

	Voltage kV	Angle		Voltage kV	Angle
Prosperina 69	68.8	− 22.8	Prosperina 69	69.1	3.9
Prosperina 230	229.8	− 19.9	Prosperina 230	235	4.9

### Salitral substation static load model

The Salitral substation ZIP static load model coefficients are shown in Table 9 on the left. The 69 kV voltage bus are shown in Table 9 on the right. Equations (17) and (18) are the ZIP static load model for Salitral substation.

**Table 9:** Salitral ZIP static load model coefficients and Voltage bus values.

Model	APC	RPC	Model	APC
Constant impedance	0.69955225	0.69177245	Maximum voltage	1.04226773
Constant current	0.24523309	0.24989994	Minimum voltage	1.00392416
Constant power	0.05521466	0.05832761	Average voltage	1.02145841

17 $$P={P}_{0}\left[0.699552{\left(\frac{V}{{V}_{0}}\right)}^{2}+0.245233\left(\frac{V}{{V}_{0}}\right)+0.055214\right]$$

18 $$Q={Q}_{0}\left[0.691772{\left(\frac{V}{{V}_{0}}\right)}^{2}+0.249899\left(\frac{V}{{V}_{0}}\right)+0.058327\right]$$

**Figure 8:**
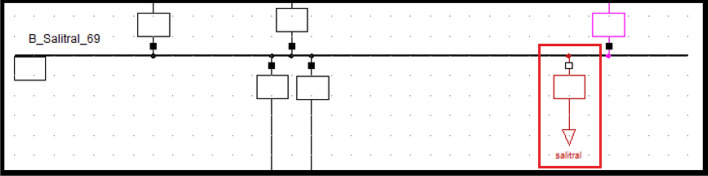
Salitral static (general) load model representation.

Figure 8 shows the PF Caraguay substation bus with the new ‘general type’ load parameters entered. This corresponds to the static load model (see red box).

The magnitude and angle of voltage on the buses after modelling the real voltage value in [pu] units of the substation are given in Table 10 Left without load model representation, right with load model representation.

**Table 10:** Magnitude and angle variation before (left) and after (right) entering Nueva Prosperina static load model.

	Voltage kV	Angle		Voltage kV	Angle
Salitral 69	69.1	− 23.6	Salitral 69	68.8	− 41.8
Salitral 138	137.4	− 20.8	Salitral 138	134.5	− 37.4
Pascuales 138	140	− 19.0	Pascuales 138	137.8	− 35.5
Pascuales 230	229.6	− 17.5	Pascuales 230	226.4	− 32.7

### Trinitatia substation static load model

The coefficients of the static load model for the Trinitalia substation are given in Table 11 on the left. The 69 kV voltage bus is shown in Table 11 on the right. Equations (19) and (20) are the ZIP static load models for the Trinitaria substation.

**Table 11:** Trinitaria ZIP static load model coefficients and Voltage bus values.

Model	APC	RPC	Model	APC
Constant Impedance	0.888706784	0.891411866	Maximum Voltage	1.0323103
Constant Current	0.108076947	0.105748492	Minimum Voltage	0.9786157
Constant Power	0.003216268	0.002839642	Average Voltage	1.00482818

19 $$P={P}_{0}\left[0.8888706{\left(\frac{V}{{V}_{0}}\right)}^{2}+0.108076\left(\frac{V}{{V}_{0}}\right)+0.003216\right]$$

20 $$Q={Q}_{0}\left[0.8914118{\left(\frac{V}{{V}_{0}}\right)}^{2}+0.105748\left(\frac{V}{{V}_{0}}\right)+0.002839\right]$$

**Figure 9:**
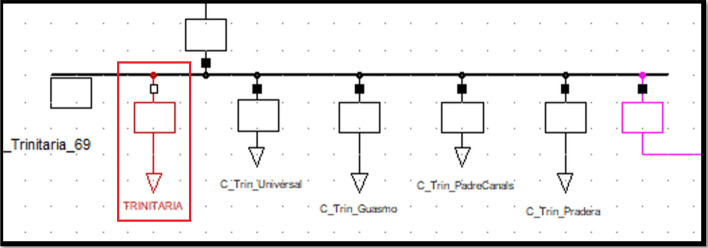
Trinitaria static (general) load model representation.

Figure 9 shows the PF Caraguay substation bus with the new ‘general type’ load parameters entered. This corresponds to the static load model (see red box).

The magnitude and angle of the bus voltage after entering the model with a real voltage value of [pu] at the substation are given in Table 12. Left without load model representation, right with load model representation.

**Table 12:** Magnitude and angle variation before (left) and after (right) entering Trinitaria static load model.

	Voltage kV	Angle		Voltage kV	Angle
Trinitaria 69	68.1	− 24.3	Trinitaria 69	68.9	2.7
Trinitaria 138	138.2	− 21.4	Trinitaria 138	140.4	3.7
Trinitaria 230	228.5	− 19.6	Trinitaria 230	233.7	5
Pascuales 230	229.6	− 17.5	Pascuales 230	234.2	5.3

## Static voltage stability analysis with load modelling

A PV curve is a graph obtained by plotting the bus voltage versus the power delivered to the load. Maximum loadability is the power value corresponding to the leading edge of the PV curve^28^. The maximum power that can be transmitted to a load depends on the availability of generated power, the power-carrying capacity of the transmission line, and the power factor of the load. The PV stability curves for six substations are shown. The PV curve shows the effect of the input load model parameters on the stability variation^29^. PV curves provide grid operators with important information about the stable operating point of the system. In addition, the system operator knows what extra load the system can tolerate without losing stability. The main consideration with figures is simplicity. Figures are large enough and have enough resolution to allow the viewer to see details.

### Trinitaria area stability

**Figure 10:**
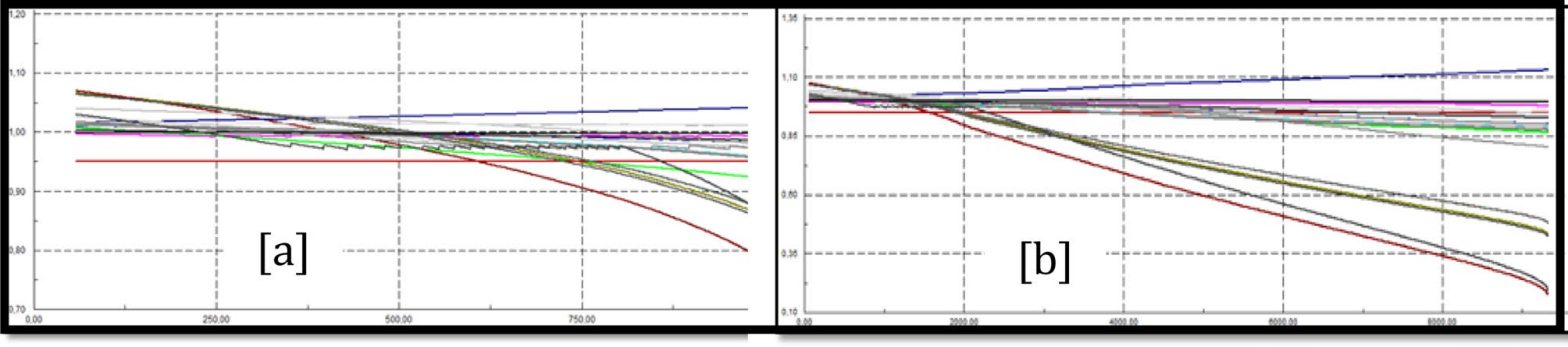
Stability PV before load model (**a**) and PV after load model (**b**) Trinitaria area.

Figure 10a shows PV Trinitaria curves before the load model and Fig. 10b shows PV Trinitaria curves after the load model.

### Caraguay 69 kV bus stability analysis

**Figure 11:**
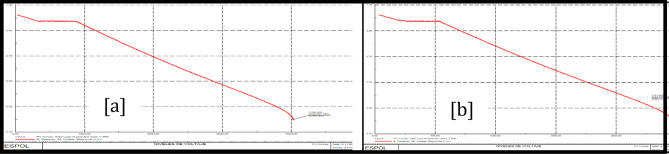
Stability PV before load model (**a**) and PV after load model (**b**) Caraguay 69 kV bus.

Figure 11a shows PV Caraguay 69 kV bus curves before the load model and Fig. 11b shows PV Caraguay 69 kV bus curves after the load model.

### Trinitaria 69 kV bus stability analysis

**Figure 12:**
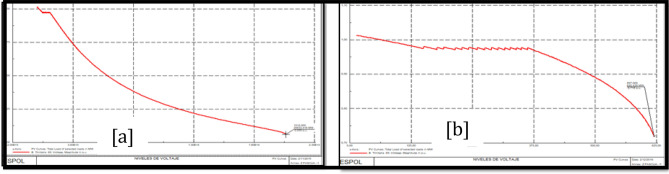
Stability PV before load model (**a**) and PV after load model (**b**) Nueva Prosperina 69 kV bus.

Figure 12a shows PV Trinitaria 69 kV bus curves before the load model and Fig. 12b shows PV Trinitaria 69 kV bus curves after the load model.

### Nueva Prosperina 69 kV bus stability analysis

**Figure 13:**
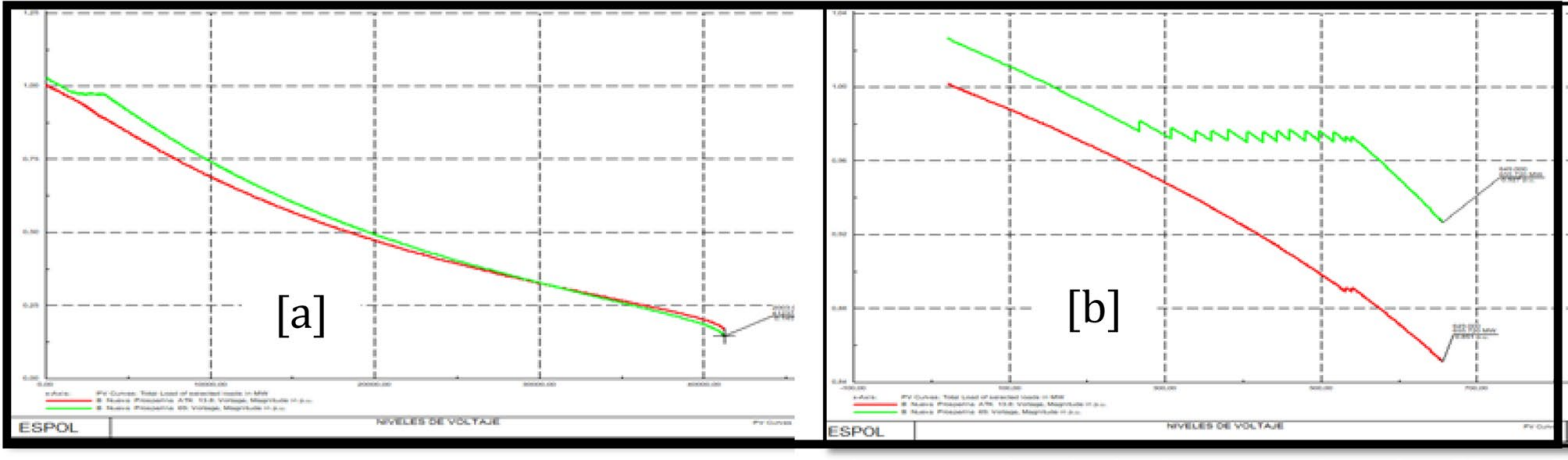
Stability PV before load model (**a**) and PV after load model (**b**) Nueva Prosperina 69 kV bus.

Figure 13a shows PV Nueva Prosperina 69 kV bus curves before the load model and Fig. 13b shows PV Nueva Prosperina 69 kV bus curves after the load model.

### Electroquil 69 kV bus stability analysis

**Figure 14:**
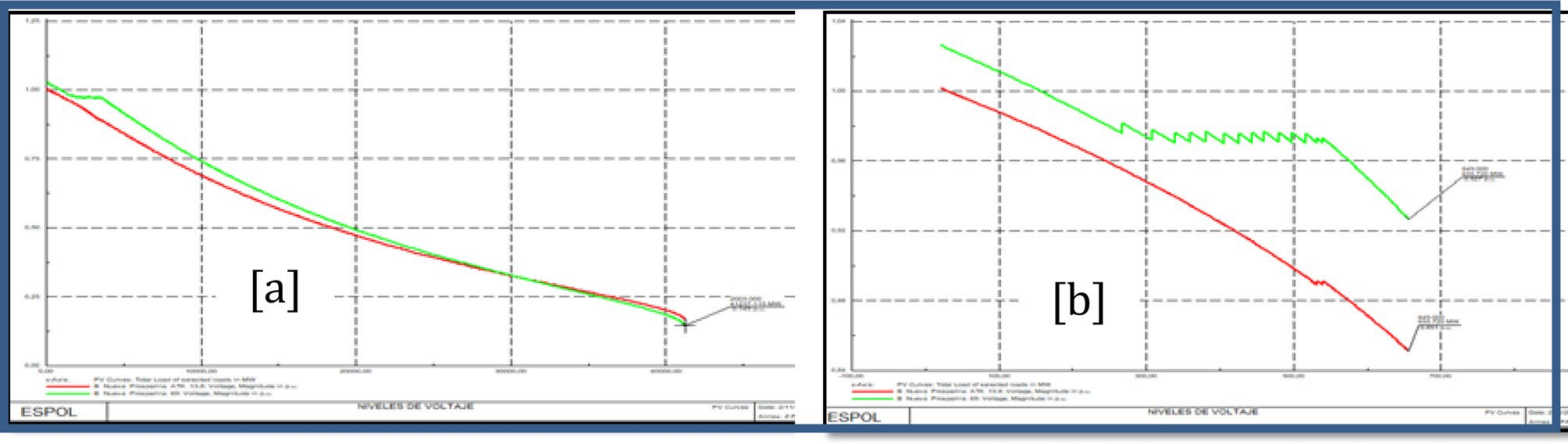
Stability PV before load model (**a**) and PV after load model (**b**) Nueva Electroquil 69 kV bus.

Figure 14a shows PV Electroquil 69 kV bus curves before the load model and Fig. 14b shows PV Electroquil 69 kV bus curves after the load model.

### Salitral 69 kV bus stability analysis

**Figure 15:**
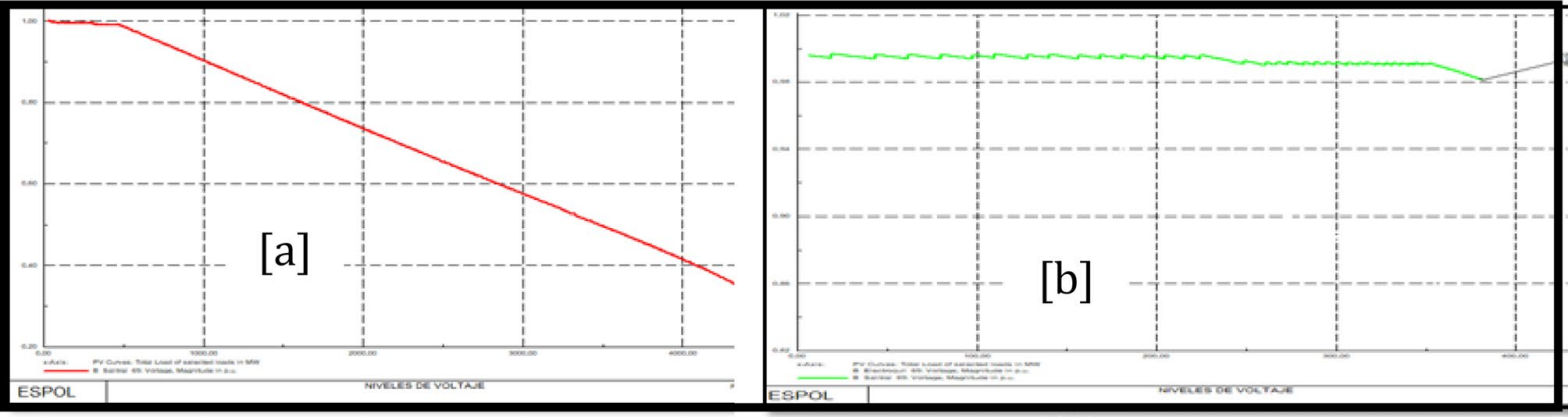
Stability PV before load model (**a**) and PV after load model (**b**) Salitral 69 kV bus.

Figure 15a shows PV Salitral 69 kV bus curves before the load model and Fig. 15b shows PV Salitral 69 kV bus curves after the load model.

### Policentro 69 kV bus stability analysis

**Figure 16:**
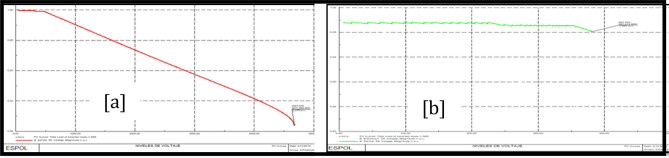
Stability PV before load model (**a**) and PV after load model (**b**) Policentro 69 kV bus.

Figure 16a shows PV Policentro 69 kV bus curves before the load model and Fig. 16b shows PV Policentro 69 kV bus curves after the load model.

### Pascuales 69 kV bus stability analysis

**Figure 17:**
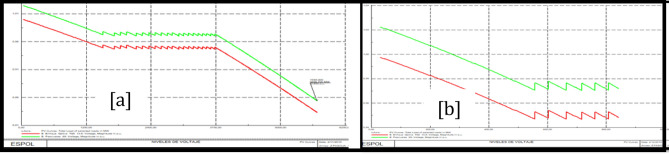
Stability PV before load model (**a**) and PV after load model (**b**) Pascuales 69 kV bus.

Figure 17a shows PV Pascuales 69 kV bus curves before the load model and Fig. 17b shows PV Pascuales 69 kV bus curves after the load model.

In summary, within the most typical variations encountered when implementing load models in the system, the stability margins are increased compared to loads previously used for power flow development in this system. In short, this study shows that the system has an excellent ability to remain operational even when the network initially presented goes down. However, as far as voltage limits are concerned, it has been observed to be close to the upper voltage limit specified for nominal operation. These results were obtained based solely on loads measured at the six substations and Electroquil in the Guayaquil area. These are not deterministic if a researcher wants to observe real failures because the load did not change at other points in the network. We do not know how this process will affect the system.

Also, by default, the PF load flow option is configured such that load dependence on voltage is not considered. That is, load is considered according to constant power model. In order for the defined static model to be considered in the load flow calculations, the ‘Consider Voltage Dependency of Loads’ option must be checked on the ‘Basic Options tab’.

## Dynamic voltage stability analysis

Load demand is the amount of power a customer requires in a particular area, under normal voltage and frequency conditions. This amount is projected based on detailed consumption information. On the other hand, the amount of electric power measured under specific voltage and frequency conditions is called load, and this value is usually differing from forecast demand. There is a trade-off between the minimum measurement time for discrimination and the effect of disturbances on the load response. If the load is heavily influenced by the disturbance, the measurement time to capture that effect should be short, but if this time is very short, there will not be enough information (sample points) to capture the real dynamics of the system. There is usually a lot of uncertainty in determining the appropriate analysis window for each case of data. This is because the system can fail and complicates the realization of Off-line sampling data identification of parameters with dynamic load. The number of sample units used for the identification greatly affects the quality of the estimates, so its choice is critical to the accuracy of the results. Long measurement times can lead to a situation that are highly susceptible to spontaneous load changes, i.e., connection or disconnection of loads, and operation of tap changer. On the other hand, a very short identification time gives a sufficiently accurate value of the transient characteristic, but not enough to calculate the time constant and steady-state value, since the load response will not yet have reached a stable level of stability.

### First perturbation model

**Figure 18:**
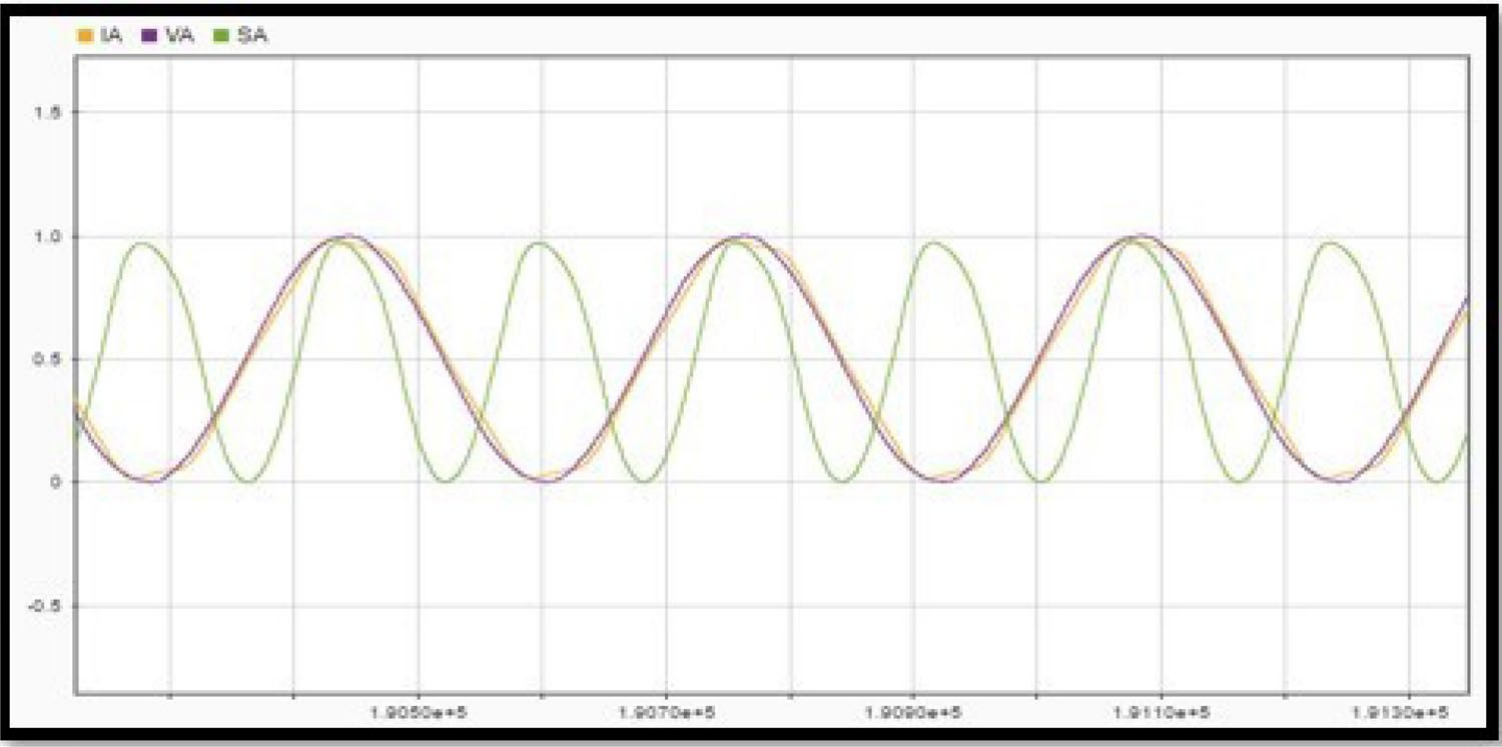
Instant current, voltage, and power.

Apparent power is obtained from the product of the instantaneous values of current and voltage. The three signals are shown in Fig. 18. A combination of sine and cosine waves with multiple frequencies can represent a periodically repeating shape as instantaneous power.

**Figure 19:**
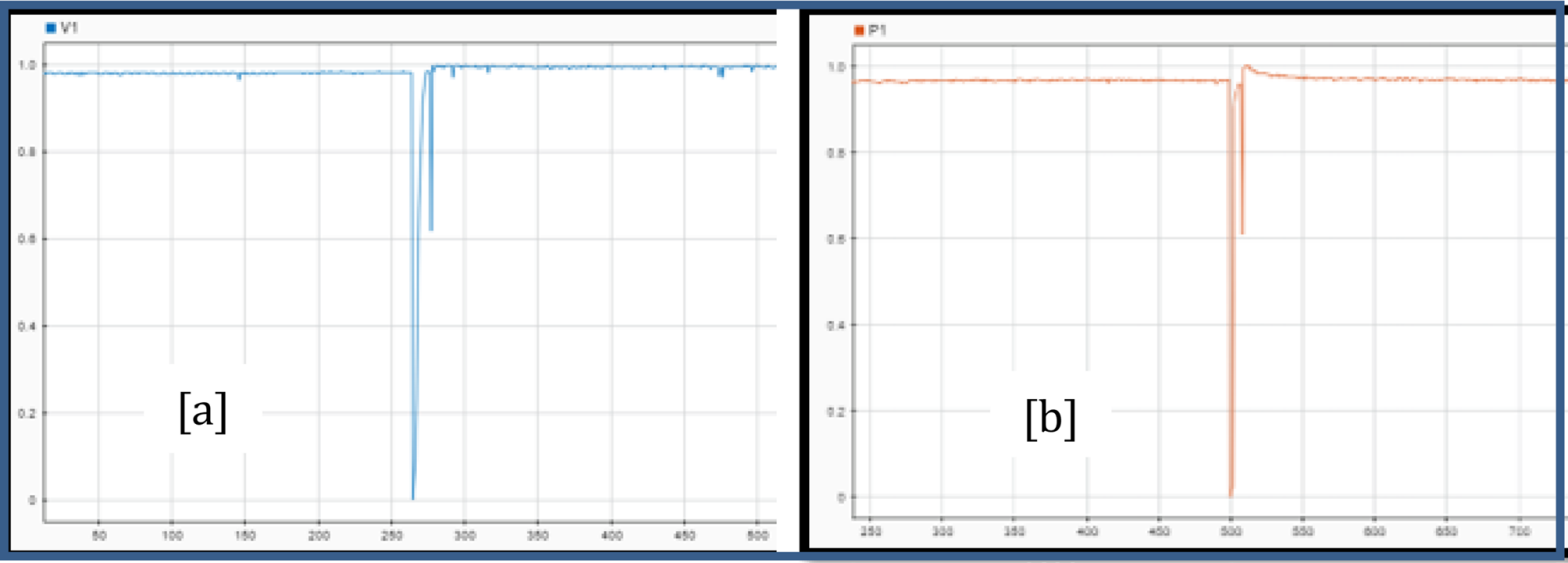
Voltage variation (**a**) and Power variation (**b**) during a disturbance at Policentro bus.

**Figure 20:**
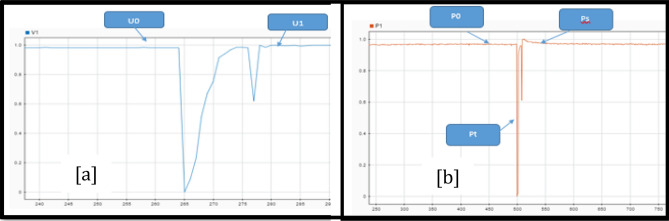
Policentro bus point selection for voltage studies (**a**) and Power studies (**b**).

Figure 19a shows the voltage and power over time during a bus fault at the Policentro substation. Considering three points of Fig. 19b and shown in Fig. 20. The voltage values are given in Table 13.

**Table 13:** Voltage and power values in the selected points of the graphs.

Voltage	Power
$\mathrm{V}0=0.95\,\mathrm{ pu}$	$\mathrm{P}0=11.53\,\mathrm{ MW}$
$\mathrm{V}1=0.54\,\mathrm{ pu}$	$\mathrm{Pt}=11.35\,\mathrm{ MW}$
	$$\mathrm{Ps}=11.42\,\mathrm{ MW}$$

Once selected, the points become parameters of the dynamic model in Eq. (3). The values U_o_ and P_0_ correspond to the voltage and power values before the disturbance; P_r_ is the active power recovery after the disturbance. T_p_ is the time required for the active power to reach 63% of the nominal power after the disturbance. That is, P_r_ = 0.63 (P_s_ − P_t_).

**Figure 21:**
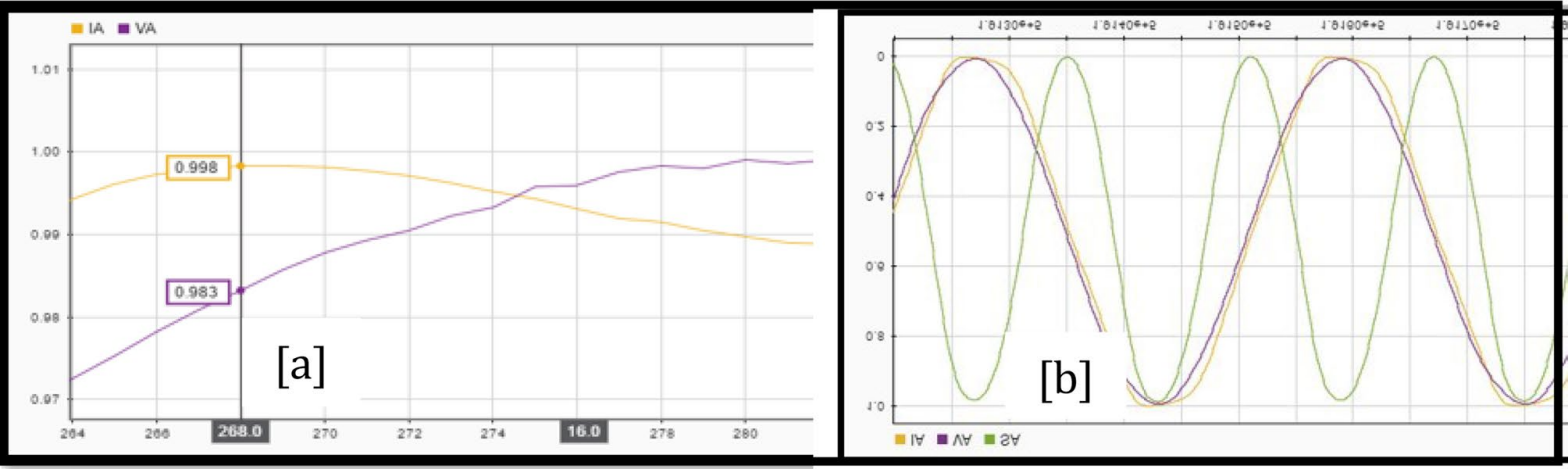
Policentro Instant power at the output (**a**) and RMS values of active demand power (**b**).

The power factor is Cosine of the resulting angle between the current and voltage in Fig. 18. The phase difference is 16 units, this value is multiplied by the factor in “Step-by-step dynamic load procedure”, which corresponds to the time interval between two consecutive data samples. That is, t = 16 $\times$ 0.000052083 = 0.8333 [ms]. The power factor is therefore 0.833, a common power factor value for distribution transformers. The instantaneous power of the system is then obtained from the product of the recorded instantaneous values of current and voltage, as shown in Fig. 21a. As seen in Fig. 21b, the waveform corresponds to a sinusoidal signal, so the RMS value of the signal is equal to the peak value divided for √2.

**Figure 22:**
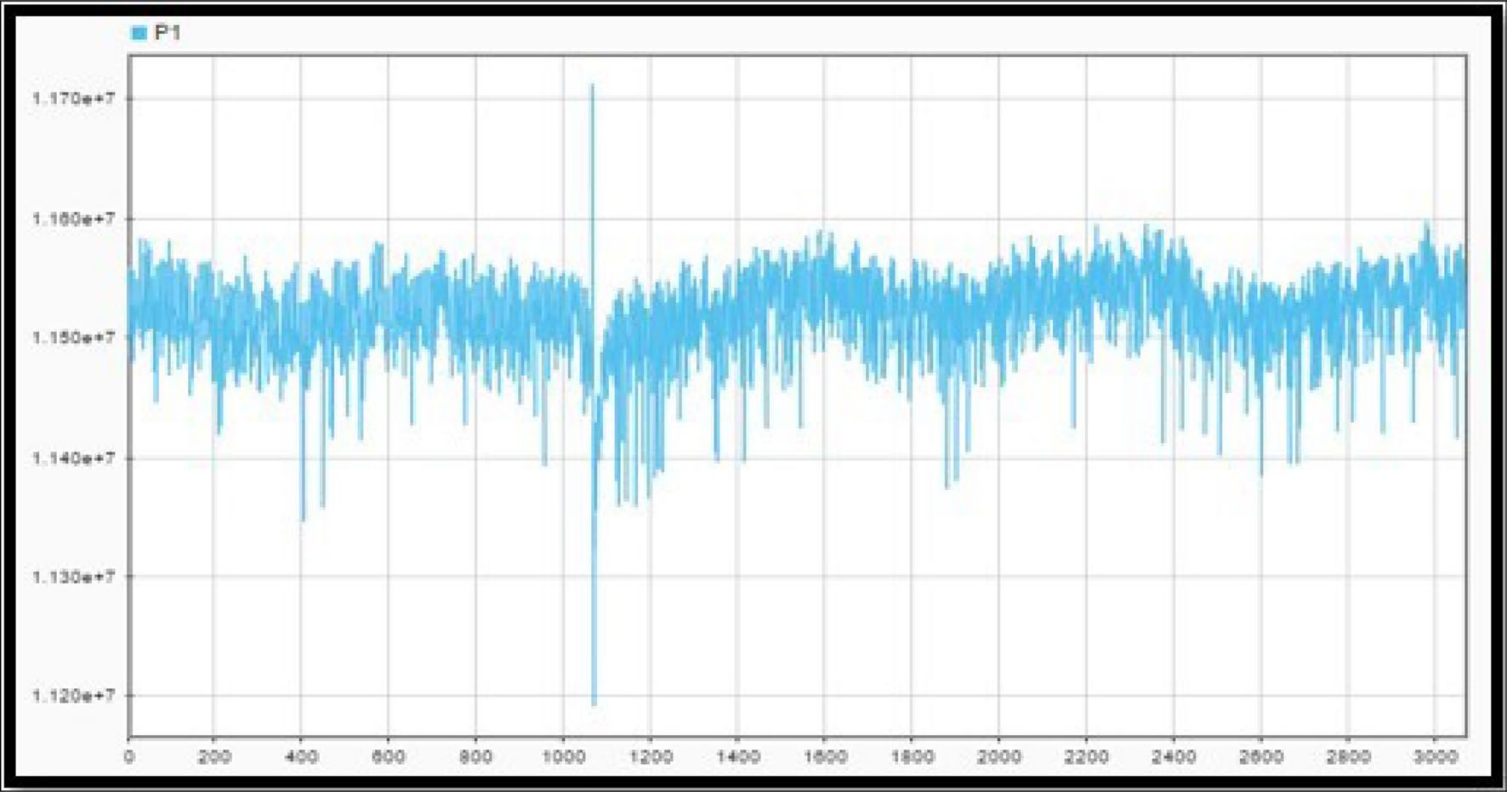
Effective values of tension.

The Routh Mean Square (RMS) of the signal (see Fig. 22) is the dynamic model of the first disturbance.

**Figure 23:**
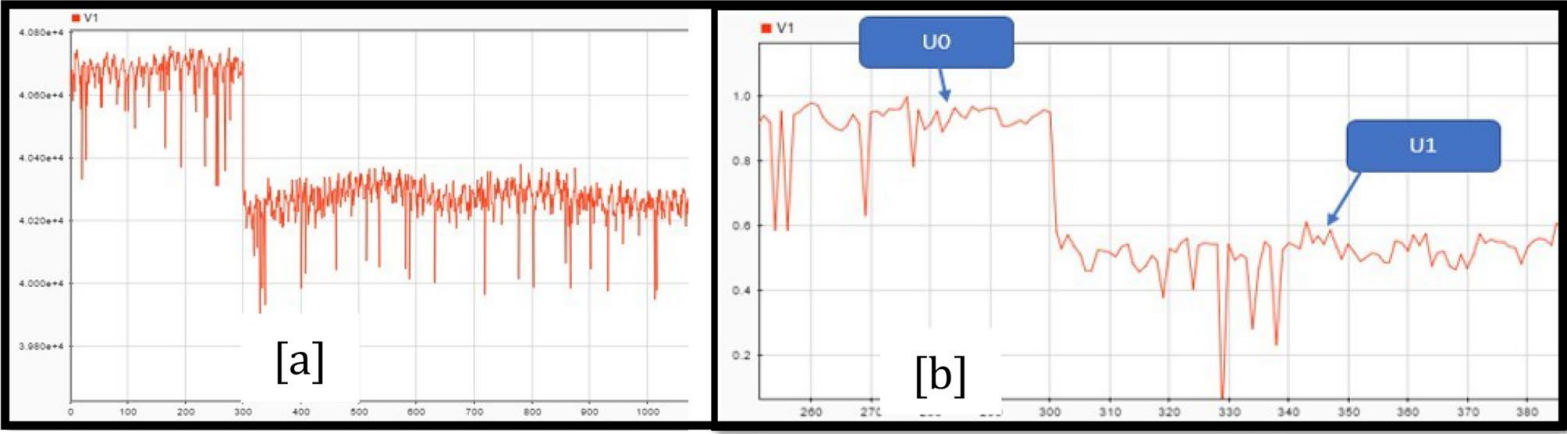
Voltage disturbance (**a**) and point selection (**b**).

To obtain a dynamic we need to analyse the portion of the curve that represents the perturbations that occur in the system and the time intervals over which the disturbance occurs. Next, three points on the graph are selected, as shown in Fig. 23 and Table 14; localized before, during and after the disturbance. That is, the first analysis point is taken before the disturbance occurs, the second point is set during the disturbance, and the third point is set when the system has reached stability and its voltage levels are within the nominal operating values.

**Table 14:** Voltage and Power values I the selected points of the graphs.

Voltage	Power
V0 = 0.95 [pu]	P0 = 11.53 [MW]
V1 = 0.54 [pu]	P1 = 11.35 [MW]
	P2 = 11.42 [MW]

The model parameters are in Eqs. (21) through (24). Where *T*_*p*_ is the power in seconds to reach 63% of the recovery value.

21 $$Pr=0.63\left(11.42-11.35\right); Pr=0.0441; Pp=0.63\left(P0\right); Pp=11.43 MW$$

22 $$Tp=1073\times \mathrm{0,000052083333}; Tp=0.05588542 s$$

The value of the *α*_*s*_ coefficient is zero because active power recovery is complete. The load under analysis is considered a constant impedance model, so its coefficient *α*_*t*_ is two.

23 $${\alpha }_{s}=0; {\alpha }_{t}=2; Uo=0.95 \; pu; Po=11.53 \; MW$$

24 $$Pl=Pr+Po{\left(\frac{U}{Uo}\right)}^{{\alpha }_{t}}; Pl=0.0441+11.53{\left(\frac{U}{0.95}\right)}^{2}$$

### Second perturbation model

The phase difference is sixteen units, multiplied by the factor in “Step-by-step dynamic load procedure”. This corresponds to the time interval between two consecutive data samples. That is, t = 16 × 0.000052083 = 0.8333 [ms]. The three points of a similar procedure of Fig. 23, for the second perturbation model can be found in Table 15.

**Table 15:** Voltage and power values in the selected points of the graphs.

Voltage	Power
$V0=0.98\, \mathrm{pu}$	$P0=12.53\, \mathrm{MW}$
$V1=0.99 \,\mathrm{pu}$	$Pt=1.03 \,\mathrm{MW}$
	$$Ps=12.5 \,\mathrm{MW}$$

The model parameters are in Eqs. (25) through (28) Where T_p_ corresponds to the value in seconds of the power to reach 63% of its recovery value.

25 $$Pr=0.63\left(12.5-1.03\right); Pr=7.2261; Pp=0.63\left(P0\right); Pp=12.4 \,\mathrm{MW}$$

26 $$Tp=507\times \mathrm{0,000052083333}; Tp=0.02640625 \,\mathrm{s}$$

The load under analysis is assumed to be a constant impedance model, so its coefficient α_t_ is two:

27 $${\alpha }_{s}=0; {\alpha }_{t}=2; Uo=0.98\, \mathrm{pu}; Po=12.53 \,\mathrm{MW};$$

28 $$Pl=Pr+Po{\left(\frac{U}{Uo}\right)}^{{\alpha }_{t}}; Pl=7.2261+12.53{\left(\frac{U}{0.98}\right)}^{2}$$

### Third perturbation model

The phase difference is eighteen units, this value is multiplied by the factor represented in “Step-by-step dynamic load procedure”. This corresponds to the time interval between two consecutive data samples, so t = 18 × 0.00005208333 = 0.9333 [ms]. Three points of similar procedure from Fig. 23 for the case of the third perturbation model can be found in Table 16.

**Table 16:** Voltage and power values in the selected points of the graphs.

Voltage	Power
$V0=0.80 \,\mathrm{pu}$	$P0=22.50 \,\mathrm{MW}$
$V1=0.90 \,\mathrm{pu}$	$Pt=19.95\,\mathrm{ MW}$
	$$Ps=22.50\, \mathrm{MW}$$

The model parameters follow in Eq. (29) through (32). Where Tp is the power in seconds to reach 63% of the recovery value.

29 $$Pr=0.63\left(22.5-19.95\right); Pr=1.6065; Pp=0.63\left(P0\right); Pp=21.51 \,\mathrm{MW}$$

30 $$Tp=439\times \mathrm{0,000052083333}; Tp=0.02286458 \,\mathrm{s}$$

The value of the *α*_*s*_ coefficient is zero because active power recovery is complete. The load under analysis is considered a constant impedance model, so the factor *α*_*t*_ is two.

31 $${\alpha }_{s}=0; {\alpha }_{t}=2; Uo=0.80 \,\mathrm{pu}; Po=22.50 \,\mathrm{MW}$$

32 $$Pl=Pr+Po{\left(\frac{U}{Uo}\right)}^{{\alpha }_{t}}; Pl=1.6065+22.50{\left(\frac{U}{0.80}\right)}^{2}$$

### Fourth perturbation model

The phase difference is six units, and this value is multiplied by the factor given in “Step-by-step dynamic load procedure”. This corresponds to the time interval between two consecutive data samples, so t = 6 × 0.00005208333 = 0.31 [ms]. Three points for a procedure similar to Fig. 23, for the case of the fourth perturbation model can be found in Table 17.

**Table 17:** Voltage and power values in the selected points of the graphs.

Voltage	Power
$\mathrm{V}0=0.95\,\mathrm{ pu}$	$\mathrm{P}0=5.78\,\mathrm{ MW}$
$\mathrm{V}1=0.55\,\mathrm{ pu}$	$\mathrm{Pt}=5.58\,\mathrm{MW}$
	$$\mathrm{Ps}=5.71\,\mathrm{ MW}$$

The model parameters follow in Eqs. (33) through (36). Where *Tp* is the power to reach 63% of the recovery value.

33 $$Pr=0.63\left(5.71-5.58\right); Pr=0.0819; Pp=0.63\left(P0\right); Pp=5.67 \,\mathrm{MW}$$

34 $$Tp=505\times \mathrm{0,000052083333}; Tp=0.02630208 \,\mathrm{s}$$

The value of the *αs* coefficient is zero because active power recovery is complete. The load under analysis is considered a constant impedance model, so its factor *αt* is two.

35 $${\alpha }_{s}=0; {\alpha }_{t}=2; Uo=0.95 \,\mathrm{pu};Po=5.78 \,\mathrm{MW}$$

36 $$Pl= 0.0819+{5.78\left(\frac{U}{0.95}\right)}^{2}$$

### Fifth perturbation model

The phase difference is fourteen units. This value is multiplied by the factor represented in “Step-by-step dynamic load procedure”. This corresponds to the time interval between two consecutive data samples, so t=14 × 0.00005208333 = 0.72 [ms]. Three points for a similar procedure of Fig. 23 for the fifth perturbation model can be found in Table 18.

**Table 18:** Voltage and power values in the selected points of the graphs.

Voltage	Power
$V0=0.73 \,\mathrm{pu}$	$P0=10.75 \,\mathrm{MW}$
$V1=0.83 \,\mathrm{pu}$	$Pt=10.45\,\mathrm{ MW}$
	$$Ps=10.77 \,\mathrm{MW}$$

The model parameters follow in Eqs. (37) through (40). Where Tp is the power in seconds to reach 63% of the recovery value.

37 $$Pr=0.63\left(10.77-10.45\right);Pr=0.2016; Pp=0.63\left(P0\right); Pp=10.71 \,\mathrm{MW}$$

38 $$Tp=562\times 0.000052083333; Tp=0.02927083\,\mathrm{ s}$$

The value of the α_s_ coefficient is zero because the active power recovery is complete. The load under analysis is considered a constant impedance model, so its coefficient α_t_ is two.

39 $${\alpha }_{s}=0; {\alpha }_{t}=2;Uo=0.73 \,\mathrm{pu};Po=10.75 \,\mathrm{MW}$$

40 $$Pl= 0.2016+{10.75\left(\frac{U}{0.80}\right)}^{2}$$

### Sixth perturbation model

The phase difference is 14 units, this value is multiplied by the factor represented in “Step-by-step dynamic load procedure”, which corresponds to the time interval between two consecutive data samples, so t = 14 × 0.00005208333 = 0.72 [ms]. Three points for a procedure similar to Fig. 23 for the case of the sixth perturbation model can be found in Table 19.

**Table 19:** Voltage and power values in the selected points of the graphs.

Voltage	Power
$\mathrm{V}0=0.90\,\mathrm{ pu}$	$\mathrm{P}0=17.80\,\mathrm{ MW}$
$\mathrm{V}1=0.86\,\mathrm{ pu}$	$\mathrm{Pt}=17.51\,\mathrm{ MW}$
	$$\mathrm{Ps}=17.84\,\mathrm{ MW}$$

The model parameters follow in Eqs. (41) through (44). Where *Tp* is the power in seconds to reach 63% of the recovery value.

41 $$Pr=0.63\left(10.84-17.51\right); Pr=0.2079; Pp=0.63\left(P0\right); Pp=17.66 \,\mathrm{MW}$$

42 $$Tp=506\times 0.000052083333; Tp=0.02635417 \,\mathrm{s}$$

The value of the *α*_*s*_ coefficient is zero because active power recovery is complete. The load under analysis is considered a constant impedance model, so its coefficient *α*_*t*_ is two.

43 $${\alpha }_{s}=0; {\alpha }_{t}=2; Uo=0.90 \,\mathrm{pu};Po=17.80 \,\mathrm{MW}$$

44 $$Pl= 0.2079+{17.80\left(\frac{U}{0.90}\right)}^{2}$$

## Discussion

Static models have been used for both static and dynamic studies. This approximation may be reasonable in some cases, although taking into account that the load expression has a significant impact on voltage stability studies, that is, it is convenient to also have dynamic models. Static models are suitable for use in static studies and can be used in dynamic studies, although this option is not recommended.

We chose the polynomial model to perform the static load models since the polynomial model corresponds to a linear combination of three other models, each of which was developed to describe a specific consumption range based on the characteristics of the most commonly relevant loads. This research uses it as an input parameter for this type of models in their PF libraries. After examining the properties of the polynomial model, the research revealed the need to know the power consumption of each sector connected to the substation for which the model was developed, so Off-line sample data were provided according to the Guayaquil sector. Also available was data for each distribution transformer connected to each substation, historical voltage levels of bus output, and total power consumption for each substation. The selection of case study is good as it covers a wide range of loads in Ecuador’s most demanded city. However, this research shows, Off-line records are generally available in some form in many, if not all, local government authorities (LA). LA should consider this type of approach to collect data from existing sources, but there is a requirement that large samples be needed so its application to be statistically valid.

It is not possible to determine dynamic load from historical records with long sampling periods. In this case, a more detailed record of load dynamics at the moment the disturbance occurs is required, which should also be adapted for load modelling identification. Therefore, the data required for the identification of dynamic load models are obtained by installing recorders that can store the exact parameters in the case of voltage or frequency. The recording device should be able to measure at a high sampling frequency (1–20 kHz) and have a storage capacity to record a complete event (up to 80 s). To avoid continuous recording, the meter must be able to detect a disturbance, start sampling minutes before the disturbance, and store it seconds after the disturbance.

A dynamic load model simulates the active and reactive power behaviour before voltage and frequency variations. By changing the transformer taps, we can generate voltage change events in real networks. However, to capture frequency events, the device must remain installed long enough to capture any disturbances that occur.

Since we are developing two types of load models, it is fair to describe the limitations found in each separately. We started with a static model because it allowed us to analyse the system in a stable state after modelling. The main limitation was data acquisition. Originally, we wanted to work with load models for buses in remote locations in the Guayaquil region, but data collection from such remote locations was not available, so sample data were selected according to historical archive data availability and in the coincidence of time between them.

The dynamic signal analyser provides a convenient tool for obtaining empirical plots of the system under investigation. The Signal Analyzer app is an interactive tool for visualizing, measuring, analysing, and comparing signals in the time domain, in the frequency domain, and in the time–frequency domain.

## Conclusions

This research uses the Off-line measuring sample data identification parameters of the digital twins mirroring, which reflects load modelling and stability analysis. It provides information from the system corresponding to the period before data analysis and processing. Off-line data samples allow analysis of different characteristics of the system at different locations and times, essentially forming an extensive database for stability studies at Guayaquil. The main drawback of Off-line measurements is that the analysis and detection of system anomalies, and control actions are not done in real-time, and therefore it is not possible to observe how the system reacts to them. In addition, the load model of the power system has different model structures and model parameters depending on the location, so the amount of data that needs to be collected off-line is large. It is not possible to generalise the system’s response to load fluctuations, since the nature of the load varies depending on the sector to which is connected.

## Data Availability

The datasets generated and/or analysed during the current study are not publicly available due to a signed non-disclosure agreement between CELEC/Transelectric and ESPOL but are available from the corresponding author on reasonable request.

## References

[1] Concordia C, Ihara S (1982). Load representation in power system stability studies. IEEE Trans. Power Appar. Syst. PAS.

[2] Taylor CW (1993). Power System Voltage Stability (Electric Power Research Institute Power System Engineering).

[3] Dzafic I, Glavic M, Tesnjak S (2004). A component-based power system model-driven architecture. IEEE Trans. Power Syst..

[4] Shi, J. H., & He, R. Measurement-based load modeling model structure. In *2003 IEEE Bologna Power Tech Conference Proceedings*, vol 2, 5 (2003). 10.1109/PTC.2003.1304621.

[5] Jones D, Snider C, Nassehi A, Yon J, Hicks B (2020). Characterising the digital twin: A systematic literature review. CIRP J. Manuf. Sci. Technol..

[6] Hartmann D, Herz M, Wever U (2018). Model Order Reduction a Key Technology for Digital Twins.

[7] Kundur P, Balu N, Lauby M (1994). Power System Stability and Control.

[8] Milanovic JV, Yamashita K, Martínez Villanueva S, Djokic S, Korunovic LM (2013). International industry practice on power system load modeling. IEEE Trans. Power Syst..

[9] IEEE Transaction on Power Systems (1993). Load representation for dynamic performance analysis (of power systems). IEEE Trans. Power Syst..

[10] Lin CJ, Chen AYT, Chiou CY, Huang CH, Chiang HD, Wang JC, Fekih-Ahmed L (1993). Dynamic load models in power systems using the measurement approach. IEEE Trans. Power Syst..

[11] Ju P, Handschin E, Karlsson D (1996). Nonlinear dynamic load modelling: Model and parameter estimation. IEEE Trans. Power Syst..

[12] Liang Y, Nwankpa CO, Fischl R, DeVito A, Readinger SC (1998). Dynamic reactive load model. IEEE Trans. Power Syst..

[13] Karlsson D, Hill DJ (1994). Modelling and identification of nonlinear dynamic loads in power systems. IEEE Trans. Power Syst..

[14] Hill DJ (1993). Nonlinear dynamic load models with recovery for voltage stability studies. IEEE Trans. Power Syst..

[15] Arif A, Wang Z, Wang J, Mather B, Bashualdo H, Zhao D (2017). Load modeling—a review. IEEE Trans. Smart Grid.

[16] Ajjarapu V (2006). Computational Techniques for Voltage Stability Assessment and Control.

[17] Wang J-C, Chiang H-D, Chang C-L, Liu A-H, Huang C-H, Huang C-Y (1994). Development of a frequency-dependent composite load model using the measurement approach. IEEE Trans. Power Syst..

[18] Caicedo G, Lozano C, Bahamón A, Arias L (2002). Modelos para estimar la demanda en sistemas de distribución [Models to estimate demand in distribution systems]. Energía Comput..

[19] Lopez K, Perez S, Rodriguez L (2016). Esquema _óptimo de deslastre de carga por baja tensión basado en Indice de estabilidad de tensión [Optimal low voltage load shedding scheme based on voltage stability index]. Ingeniería Investig..

[20] Google earth V 7.3.6.9326. (December 13, 2022). Guayaquil, Ecuador. 2° 11’ 33.09” S, 79° 52’ 45.16” W, La Rotonda Grid. Eye alt 22.03 km. http://www.earth.google.com. Accessed 23 Dec 2022.

[21] Madlool N, Saidur R, Rahim N, Kamalisarvestani M (2013). An overview of energy savings measures for cement industries. Renew. Sustain. Energy Rev..

[22] Kubule A, Zogla L, Rosa M (2016). Resource and energy efficiency in small and medium breweries. Energy Proced..

[23] Hajagos LM, Danai B (1998). Laboratory measurements and models of modern loads and their effect on voltage stability studies. IEEE Trans. Power Syst..

[24] Bircan M, Durusu A, Kekezoglu B, Elma O, Selamogullari US (2020). Experimental determination of zip coefficients for residential appliances and zip model-based appliance identification: The case of ytu smart home. Electr. Power Syst. Res..

[25] Omata T, Uemura K (1998). Aspects of voltage responses of induction motor loads. IEEE Trans. Power Syst..

[26] Sadeghi, M., & Abdollahi sarvi, G. Determination of zip parameters with least squares optimization method. In *2009 IEEE Electrical Power Energy Conference (EPEC)*, 1–6 (2009). 10.1109/EPEC.2009.5420883.

[27] Kulkarni AV, Gao W, Ning J (2010). Study of power system load shedding scheme based on dynamic simulation. IEEE PES T D.

[28] Morison G, Gao B, Kundur P (1993). Voltage stability analysis using static and dynamic approaches. IEEE Trans. Power Syst..

[29] Wang, S., Lee, S., Wu, Y., & Wu, C. Analysis of load characteristics in power systems based on fuzzy modeling. In *2012 Proceedings of SICE Annual Conference (SICE)*, 1067–1070 (2012).

